# Genetic dissection of branch architecture in oilseed rape (*Brassica napus L*.) germplasm

**DOI:** 10.3389/fpls.2022.1053459

**Published:** 2022-10-28

**Authors:** Ying Wang, Kaixuan Wang, Tanzhou An, Ze Tian, Xiaoling Dun, Jiaqin Shi, Xinfa Wang, Jinwu Deng, Hanzhong Wang

**Affiliations:** ^1^ Oil Crops Research Institute of the Chinses Academy of Agricultural Sciences, Key Laboratory of Biology and Genetic Improvement of Oil Crops, Ministry of Agriculture, Wuhan, China; ^2^ Hubei Hongshan Laboratory, Wuhan, China

**Keywords:** oilseed rape, branch angle, GWAS, transcriptome, candidate gene

## Abstract

Branch architecture is an important factor influencing rapeseed planting density, mechanized harvest, and yield. However, its related genes and regulatory mechanisms remain largely unknown. In this study, branch angle (BA) and branch dispersion degree (BD) were used to evaluate branch architecture. Branch angle exhibited a dynamic change from an increase in the early stage to a gradual decrease until reaching a stable state. Cytological analysis showed that BA variation was mainly due to xylem size differences in the vascular bundle of the branch junction. The phenotypic analysis of 327 natural accessions revealed that BA in six environments ranged from 24.3° to 67.9°, and that BD in three environments varied from 4.20 cm to 21.4 cm, respectively. A total of 115 significant loci were detected through association mapping in three models (MLM, mrMLM, and FarmCPU), which explained 0.53%-19.4% of the phenotypic variations. Of them, 10 loci were repeatedly detected in different environments and models, one of which *qBAD.A03-2* was verified as a stable QTL using a secondary segregation population. Totally, 1066 differentially expressed genes (DEGs) were identified between branch adaxial- and abaxial- sides from four extremely large or small BA/BD accessions through RNA sequencing. These DEGs were significantly enriched in the pathways related to auxin biosynthesis and transport as well as cell extension such as indole alkaloid biosynthesis, other glycan degradation, and fatty acid elongation. Four known candidate genes *BnaA02g16500D* (*PIN1*), *BnaA03g10430D* (*PIN2*), *BnaC03g06250D* (*LAZY1*), and *BnaC06g20640D* (*ARF17*) were identified by both GWAS and RNA-seq, all of which were involved in regulating the asymmetric distribution of auxins. Our identified association loci and candidate genes provide a theoretical basis for further study of gene cloning and genetic improvement of branch architecture.

## Introduction

Plant architecture refers to the spatial distribution pattern of above-ground parts (mainly including plant height, branch/tiller angle, number and length of tillers/branches, and organ morphology), and it is influenced by both genetic and environmental factors (Wang and Li, 2006; Wang et al., 2018). For crops, the ideal plant architecture is important for yield improvement (Donald, 1968; Wang and Li, 2005; Jin et al., 2008; Jiao et al., 2010). The branch angle or tiller angle plays a vital role in plant adaptation to high-density cultivation, and it is subjected to strong selection during crop improvement (Wang et al., 2021; Zheng et al., 2022).

Oilseed rape (*Brassica napus L*., 2n=38, genome AACC) is a globally important crop that provides vegetable oil for human consumption, biodiesel for industry, oilcake for breeding, and fertilizer for soil (Wang et al., 2011; Zheng et al., 2022). Oilseed rape branch architecture such as branch angle (BA)and branch dispersion degree (BD) are key morphological traits that have a significant effect on plant density, collapse resistance, and disease resistance, especially on crop yield (Wang and Li, 2008; Zheng et al., 2021). Plants with small branch angles or compact branch architecture are more suitable for mechanized harvest and yield improvement. Clarifying the genetic basis underlying branch angle and branch dispersion degree in oilseed rape will be of great value for plant architecture improvement.

Genome-wide association study (GWAS) based on linkage disequilibrium (LD) between molecular markers and target genes has been widely used to analyze quantitative traits, discover loci associated with phenotypic variation, and identify candidate genes and molecular markers for breeding application (Xiao et al., 2017; Tibbs Cortes et al., 2021). The *Brassica* 50K single nucleotide polymorphism (SNP) Infinium array allows revealing the genetic basis of agronomic traits at the DNA level for oilseed rape (Chalhoub et al., 2014; Xiao et al., 2021). In recent years, association mapping has been extensively used to study a variety of traits of oilseed rape, including plant height, oil content, and flowering time (Wang et al., 2011; Li et al., 2021; Liu et al., 2021; Vollrath et al., 2021). In addition, transcriptomic sequencing provides a high-throughput approach to identifying differentially expressed genes (DEG) at the whole genome level. The combination of transcriptome and genome-wide association analysis has been confirmed to be an effective method for identifying causative or candidate genes regulating complicated quantitative traits (Li et al., 2021; Liu et al., 2021; Lv et al., 2022; Tan et al., 2022).

Linkage mapping, association mapping, and transcriptomic analysis have been widely used for the study of branch angle of oilseed rape (Liu J, et al., 2016; Sun et al., 2016; Wang H. et al., 2016; Cheng et al., 2017; Li et al., 2017; Shen et al., 2018; Wang et al., 2019; Zhao et al., 2021). Using association mapping, more than 100 association loci and multiple candidate orthologues such as *LAZY1*, *SPL14*, *GH3*, *PIN1*, *TAC1*, *SGR1/2/3/5* related to branch angle have been identified from three different natural populations in oilseed rape (Li et al., 2017; Liu J. et al., 2016; Sun et al., 2016). By QTL mapping, about 30 QTLs and multiple candidate homologs related to branch angle including *GH3*, *SUAR*, *TPR2/4*, *YUCCA1*, *ARF10*, *CPK24*, and *VAMP714* have been detected from different linkage populations in oilseed rape (Shen et al., 2018; Wang et al., 2019; Zhao et al., 2021). Through transcriptome analysis, multiple DEGs related to auxin and brassinolide pathways have been identified in the study of branch angle in *B.napus* (Cheng et al., 2017). However, few of these studies have combined both GWAS and RNA-seq to explore the genetic basis of branch architecture in oilseed rape. In addition, the related genes and underlying mechanisms are largely unclear.

In this study, we observed the dynamic changes of branch angle (BA) throughout branch development period from branch emergence to maturity, and chose the stage after flowering for BA measurement. We introduced branch dispersion degree (BD) to evaluate the compactness of branch architecture. The branch joints of extreme materials were observed at the cytological level. To maximize the QTL detection efficiency and minimize false positives, GWAS of BA and BD was performed based on three models MLM, mrMLM, and FarmCPU. Furthermore, a transcriptome analysis was performed to investigate the adaxial and abaxial sides of the extreme branches. A total of 115 loci and 4 candidate genes were identified by combing GWAS and RNA-seq, of which *qBAD.A03-2* was further verified as a stable QTL using a segregated secondary population. Our findings will provide a new perspective for further gene cloning and genetic improvement of branch architecture in oilseed rape.

## Materials and methods

### Plant accessions and trait measurements

A total of 327 diverse oilseed rape accessions including 102 spring, 191 semi-winter, and 34 winter ecotypes were used in this study. Throughout three growing seasons, field experiments were performed in six different environments. These 327 accessions were grown at Yangluo (114.50°E, 30.38°N) in Hubei province during the growing seasons of 2018, 2019, and 2020. The location Yangluo in these 3 years was referred to as 3 environments E1, E2, and E3. The field experiments carried out at Wuhan (113.68°E, 30.58°N) and Xiangyang (112.14°E, 32.04°N) in Hubei province, and at Haidong (102.10°E, 36.50°N) in Qinghai province in 2020 growing season were referred to as another 3 environments E4, E5, and E6. The field experiments of each accession were performed in three block replicates. Field management followed standard agriculture practices. The branch architecture was measured approximately two weeks after the final flowering stage. The third and fourth branches from the plant top were chosen for branch angle measurements, since the angles of these two branches were reported to exhibit the highest correlation with the average angle of all the branches in an individual plant (Sun et al., 2016).

**Figure 1:**
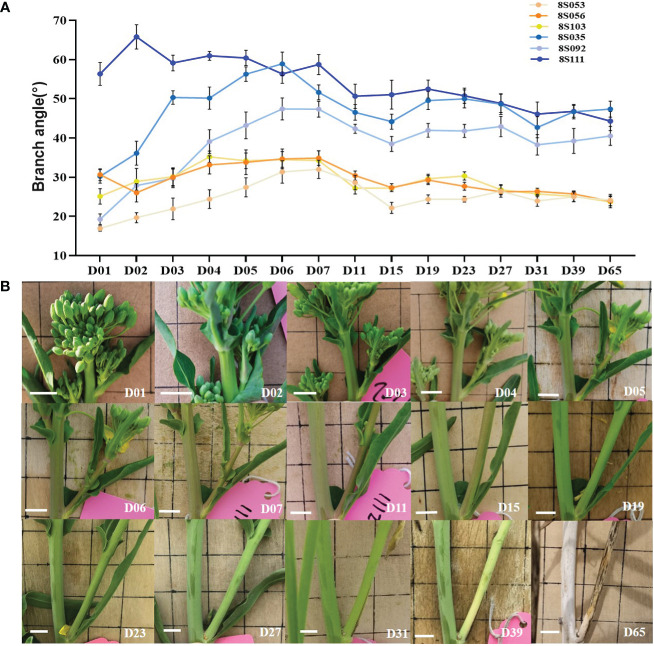
Dynamic observation of branch angle formation. **(A)** The branch angle of three compact (8S053, 8S056, 8S103, orange lines) and loose accessions (8S035, 8S092, 8S111, blue lines) were observed from branch emergence to maturity (D01- D65). **(B)** The branch angle dynamic variation of the compact accession (8S056) visually. D01 is the abbreviation of Day01, which means the first day of branch emergence.

In this study, we investigated two traits branch angle (BA) which refers to the angle between the branch base and the main stem, and the branch dispersion degree (BD) to evaluate branch architecture in oilseed rape (**Figure S1**). The average branch length of all 327 plants was about 50 cm. BD was defined as the distance between the branch and stem at the point 25 cm (about the middle point of branches) far from the branch junction (**Figure 1**). BD of the third and fourth branches from the plant top was measured manually. For each field block, at least five individuals were sampled and measured. The phenotypic variation and correlation analysis of BA and BD were performed using SAS software (Version 9.3, SAS Institute Inc., Cary, NC, US). The broad-sense heritability was calculated using the previously reported formula (Liu S et al., 2016). Pearson’s correlation analysis of BA and BD was performed using Origin Pro software.

### Dynamic changes of BA and cytological observation

The third branches of three loose (8S111, 8S092, 8S035) and three compact accessions (8S056, 8S103, 8S053) were chosen for dynamic branch angle observation from branch emergence to branch maturity. The first day of branch emergence was defined as D01, and the branch angle was photographed. At least six individual plants of each accession were examined, and their branch angles in the photos were measured using Image J software.

The third branch junction in the stable stage (from day 15 to 65 post-branch emergence) was sampled and fixed in Carnoy’s solution containing 70% ethanol and 30% acetic acid for 12 h. Following ethanol serial dehydration, samples were then embedded in paraffin wax. Histological sections were prepared using a Leica HistoCore AUTOCUT (Leica, Germany). Sections were stained with Periodic Acid-Schiff (PAS) and photographed using a SOPTOP EX30 microscope (SOPTOP, China) (Schaart et al., 2004).

### Genome-wide association study

The *Brassica* 50K SNP array was used for genotyping of the association population. The Illumina Bead Studio genotyping software (http://www.Illumina.com/) was used to screen effective SNP with the following parameters: missing rate ≤ 0.2, heterozygous rate ≤ 0.2, and minor allele frequency > 0.05. A total of 21,242 SNPs were screened out for subsequent analysis. The probe sequences of these SNP markers were aligned to the *B. napus Darmor-bzh* reference genome to identify their physical positions (Chalhoub et al., 2014). Detailed information on SNP genotypes was obtained from the previous report (Ibrahim et al., 2021).

Three models were used for the association study, including the mixed linear model (MLM) based on both structure matrix and relative kinship (Liu J. et al., 2016), multi-locus random-SNP-effect mixed linear model (mrMLM) (Li et al., 2022), and fixed and random model circulating probability unification model (FarmCPU) (Yin et al., 2021). Both MLM and FarmCPU were implemented by rMVP in R package. The mrMLM was conducted by mrMLM in R package. The threshold for the significantly associated SNP markers was set as P < 4.71 × 10^−5^ [P = 1/21242, −log10 (P) = 4.33]. To estimate the total phenotypic variation explained by the significant SNPs in the best-fitting multiple regression model, the best linear unbiased estimators (BLUEs) of BA and BD traits were respectively calculated using lme4 and lsmean in R package. The obtained BA-BLUE and BD-BLUE values were used for GWAS.

### RNA-seq analysis of branch angle and qRT–PCR validation

Two large branch angle accessions 8S111 (large 1, L1) and 8S092 (large 2, L2), and two small branch angle accessions 8S103 (small 1, S1) and 8S056 (small 2, S2) were selected for RNA-seq. Tissue samples were collected from the adaxial side (LU1, LU2, SU1, SU2) and the abaxial side (LD1, LD2, SD1, SD2) of the third branch at the beginning of flowering. The samples from 10 individual plants were mixed into one biological replicate with three biological replicates per sample. Total RNA was extracted with RNA prep Pure Plant Kit (Tiangen, China) according to the instruction. The cDNA libraries were constructed and subjected to Illumina sequencing using an Illumina NovaSeq6000 platform. The adapters and low-quality data were removed from the raw data. The resulting clean data were aligned to the reference genome of *Brassica napus L*. (http://cbi.hzau.edu.cn/bnapus/). The transcript abundance was determined based on the fragments per kilobase of the exon model per million mapped reads (FPKM). The differentially expressed genes (DEGs) were identified using the DESeq2 software with the thresholds of Padjust ≤ 0.05 (adjusted P for the false discovery rate, FDR) and |log2 (fold change)| ≥ 1. The k-mean clustering was performed by MeV_4_9_0 software. Gene ontology (GO) and Kyoto Encyclopedia of Genes and Genomes (KEGG) enrichment analysis were performed using the ClusterProfiler package in R.

To verify the accuracy of the RNA-seq data, four genes (*BnaA03g15370D*, *BnaA03g22230D*, *BnaC07g29140D*, *BnaC09g39930D*) randomly were chosen from the DEGs for quantitative real-time PCR (qRT-PCR). The primers’ information was presented in **Table S1**. The qRT-PCR was conducted with the SYBR qPCR Master Mix kit (Vazyme) using the instrument CFX96 (BIO-RAD). For each sample, there were three biological replicates. The 2-^ΔΔCT^ method was used to calculate the relative expression of the target genes in B. napus with *ACTIN7* as an internal control.

### Identification of candidate genes

The genes within 500 kb upstream and downstream of the significant SNP loci were selected as putative candidate genes based on the decay of linkage disequilibrium (LD) (Li S. et al., 2020). The *B. napus* reference genomes were used to align the QTL intervals (http://www.genoscope.cns.fr/projet_CCM/cgi-bin/gbrowse/bnapus-bzh/). Candidate genes were identified based on the reliable association loci screened by association analysis, reported branch angle genes, and differentially expressed genes detected by transcriptomic analysis.

## Results

### Dynamic changes of branch angle and cytological observation

We continuously observed the representative third branch of three loose (8S035, 8S092, and 8S111) and three compact accessions (8S053, 8S056, and 8S103) from branch emergence to maturity. The branch angles of all observed accessions increased during the early stage (D01-D07), and decreased slightly after one week (D07-D15), then became stable, with the increasing branch lignification and stem rigidity (D15-D65) (**Figure 1A**). **Figure 1B** showed the dynamics from branch emergence to maturity of 8S056 accession. There was a significant difference (P=2.3E-10) in branch angles between loose (47.3°) and compact (27.2°) accessions during the stable stage. Based on the above observations, the period after flowering (nearly two weeks after the branch growing out) was selected as the optimal period for population trait examination.

**Figure 2:**
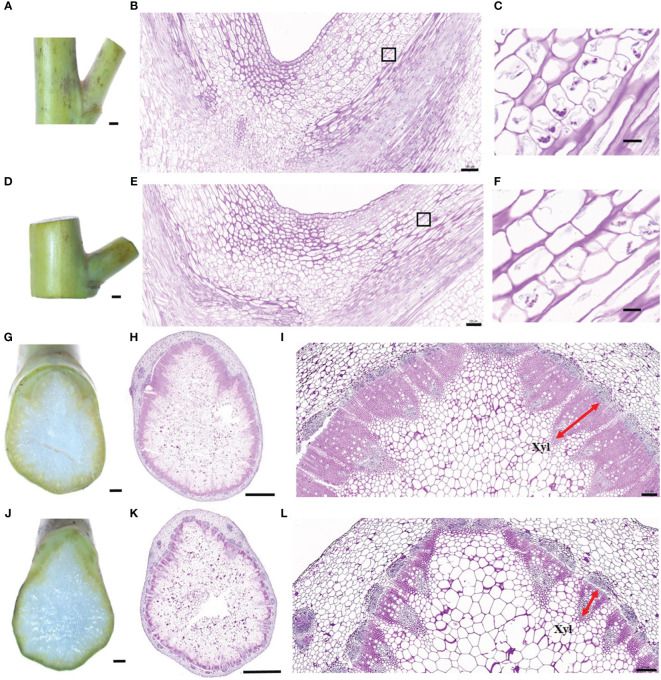
Cytological observation of branch joints between compact and loose accessions. **(A)** Branch joint of the compact accession (8S056). **(B)** Longitudinal section of branch joint from the compact accession. **(C)** Enlarged view of a black rectangular box indicated region of **(B)**. **(D)** Branch joint of the loose accession (8S092). **(E)** Longitudinal section of branch joint from the loose accession. **(F)** Enlarged view of the black rectangular box indicated region of **(E)**. **(G–I**) The bottom horizontal section of the branch joint in the compact accession (8S056). **(J–L)** The bottom horizontal section of the branch joint in the loose accession (8S092). The red arrow indicates the xylem. Bars = 1 mm in **(A, D, G, J)**, 2 mm in **(H, K)**, 500 μm in **(B, E, I, L)**, and 20 μm in **(C, F)**.

To further explore the reason for the branch angle difference between the six accessions from a cytological perspective, the paraffin sections of the branch junction were observed under the microscope (**Figures 2A–F**). The results of longitudinal cutting showed that the compact accession had more amyloplasts (**Figures 2B, C**) in endodermis than the loose accession (**Figures 2E, F**), suggesting that compact accession might be more sensitive to gravity. Additionally, a branch horizontal cutting at the point where the branch connected to the stem showed that the vascular bundle of the compact accession had larger xylem (**Figures 2G–I**) than the loose accession (**Figures 2J–L**), indicating that the xylem size difference might be responsible for branch angle difference between compact and loose accessions.

### Phenotypic variation of branch architecture in oilseed rape germplasm

**Figure 3:**
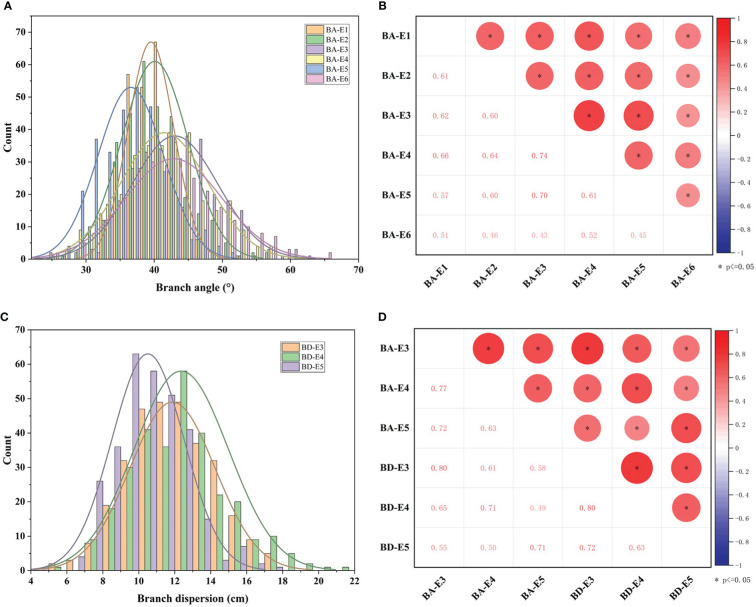
The phenotypic distribution and correlation of BA and BD across 327 accessions in environments. **(A)** The distribution of BA in six environments (BA-E1, BA-E2, BA-E3, BA-E4, BA-E5, and BA-E6). **(B)** The correlation analysis of BA between six environments. **(C)** The distribution of BD in three environments (BD-E3, BD-E4, and BD-E5). **(D)** The correlation analysis of BD and BA between E3, E4, and E5 environments. * represents the significance level of p ≤ 0.05.

To further explore the phenotypic variation of branch architecture in germplasm, two traits BA and BD of 327 accessions were investigated. The results showed that in six environments (E1-E6), the branch angle of 327 accessions exhibited normal and near-normal distribution (**Figure 3A**) with a wide variation from 24.3° to 67.9° and an average value of 40.5° (**Table S2A**). The coefficient of variation ranged from 8.6% to 16.5% in the six environments (**Table S2A**), indicating abundant genetic variations in the population. All the branch angles displayed a significant correlation between environments with correlation coefficients ranging from 0.43 to 0.74 (**Figure 3B**). The broad-sense heritability (H^2^) was calculated as 88.18% (**Table S2A**), indicating that branch angle in the population was relatively less influenced by environments, and was predominantly influenced by genetic factors.

In the three environments (E3, E4, E5), the BD varied from 4.20 cm to 21.4 cm, with an average of 10.5 ± 2.0 cm, 11.9 ± 2.4 cm, and 12.4 ± 2.6 cm, respectively. The coefficient of variation ranged from 19.0% to 21.3% in the three environments (**Figure 3C**; **Table S2B**). The broad-sense heritability (H^2^) for BD was 88.54% (**Table S2B**), suggesting that the degree of branch dispersion was also influenced primarily by genetic factors. As expected, the BD of 327 accessions showed a significant positive correlation (P < 0.05) among three environments with correlation coefficients of 0.63, 0.72, and 0.80 (**Figure 3D**).

Moreover, a significant positive correlation (P<0.05) was observed between BA and BD in the same environment with high correlation coefficients of 0.80, 0.71, and 0.71, respectively (**Figure 3D**). This suggested that the two traits were highly correlated, and thus they were suitable for the evaluation of branch architecture.

### Genome-wide association study of BA and BD

**Figure 4:**
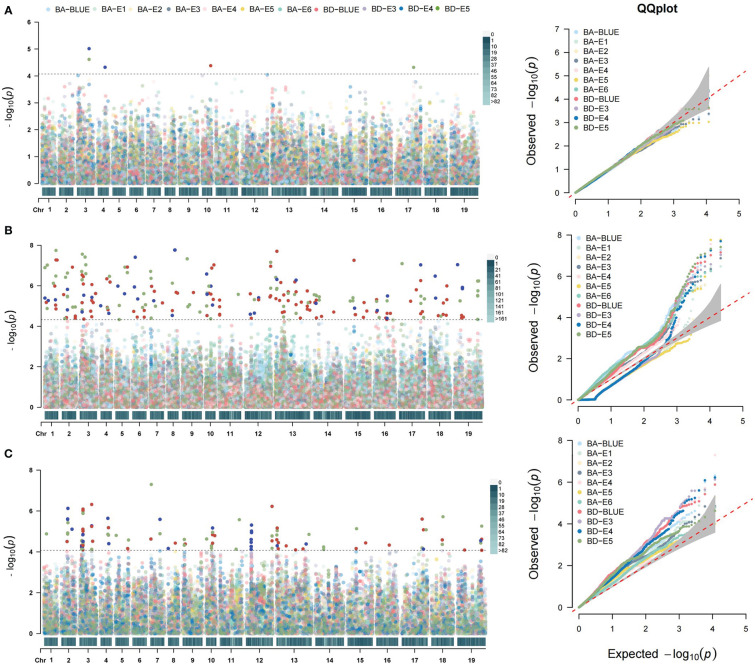
Genome-wide association analysis of BA and BD. **(A)** Manhattan (left) and Q-Q plots (right) of BA and BD using MLM model. **(B)** Manhattan and Q-Q plots of BA and BD using mrMLM model. **(C)** Manhattan and Q-Q plots of BA and BD using FarmCPU model. The different colors distinguished the different environments, as shown in the legends. The horizontal dotted line indicates the significance threshold [–log10 (*p*) = 4.3].

To further explore the genetic basis of branch architecture in oilseed rape, we performed a genome-wide association study of the above-mentioned association panel of 327 accessions. The BA in six environments (BA-E1, BA-E2, BA-E3, BA-E4, BA-E5, BA-E6), BD in three environments (BD-E3, BD-E4, BD-E5), and their best linear unbiased estimators (BA-BLUE, BD-BLUE) were employed for association analysis using three models MLM, mrMLM, and FarmCPU. Briefly, MLM detected 5 significant SNPs, three of which were only for BA, one for BD, and one for both traits (**Figure 4A**; **Table S3**). These 5 SNPs were distributed on chromosomes A03, A04, A10, C03, and C07, explaining 1.50% to 3.45% phenotypic variation. mrMLM identified 146 significant SNPs, of which 66 were found only for BA, 71 were found only for BD, and 9 were found for both traits. They were distributed on all the chromosomes except C01, and the explained phenotypic variation ranged from 0.53% to 19.4% (**Figure 4B**; **Table S3**). FarmCPU detected 53 significant SNPs, of which 16 were found only for BA, 32 were only for BD, and 5 were for both traits (**Figure 4C**; **Table S3**) These significant SNPs detected by FarmCPU were distributed on most chromosomes except A06 and C04 (**Figure 4C**; **Table S3**). Further, the GWAS results obtained from the three models were also compared. Two significant SNPs, Bn-A03-p22132555 and chrA10_14922813 were commonly detected by all three models, which explained 5.11% and 4.69% of the phenotypic variation, respectively. Four significant SNPs detected by both MLM and mrMLM including Bn-A03-p22132555, seq-new-rs25319, seq-new-rs26844, and chrA10_14922813. In total, 15 significant SNPs were detected by both mrMLM and FarmCPU models, of which only one (chrA03_6002385) was related to both traits. Seven significant SNPs were identified by both MLM and FarmCPU models, of which one SNP (Bn-A03-p22132555) was associated with both traits (**Table S3**).

In combination with BA and BD in all the environments and three models, these significant SNPs were integrated into 115 association loci. Compared with the reported QTLs related to branch angle, our detected 42 QTLs overlapped with the corresponding physical intervals previously reported (**Table S4**). Therefore, the remaining 73 QTLs were the new QTLs regulating the branch architecture of rapeseed (**Table S4**). Of 115 QTLs, 40 and 49 QTLs respectively controlled BA and BD, and only 26 (22.6%) were responsible for both traits, suggesting the different genetic control over the two traits (**Table S4**). Of 115 QTLs, 10 were commonly detected in more than two environments and models, and thus they represented stable QTLs and were selected for further study (**Table 1**).

**Table 1:** 10 stable association loci of branch architecture in oilseed rape.

Associated loci	Physical interval (bp)	Environment	Method	'-log10(P)'	r2 (%)	Homologous to known BA genes	Involving in known BA pathways	DEGs		Reference
								LD vs SD	LU vs SU	
*qBAD.A01-3*	17,371,197-19,439,099	BA-E1, BA-E5, BA-E6, BA-BLUE, BD-E4	mrMLM	4.54-6.48	1.67-6.52		BnaA01g27570D (EBP)	\	\	New
							BnaA01g26080D (Gibberellin-regulated family protein)	\	\
*qBAD.A02-1*	9,090,101-11,735,555	BA-E1, BD-E3, BD-E4, BD-E5, BD-BLUE	mrMLM, FarmCPU	4.51-10.1	4.04-15.9	BnaA02g16500D (PIN1)		\	\	Liu J, et al, 2016
							BnaA02g15660D (VND7)	\	\
							BnaA02g15710D (TCP )	\	\
							BnaA02g15980D (SAUR)	\	\
							BnaA02g16580D (BEE3)	\	\
*qBAD.A03-1*	3,273,797-4,324,749	BA-E3, BA-E4, BA-BLUE, BD-E3,BD-E4, BD-E5, BD-BLUE	mrMLM, FarmCPU	4.53-7.74	1.82-6.46	BnaA03g10430D (PIN2)		\	\	Shen et al., 2018
							BnaA03g08500D (VAMP714)	Upregulated	Upregulated	Liu S, et al., 2016
							BnaA03g08090D (SAUR)	\	\	Wang et al., 2019
							BnaA03g08100D (SAUR)	\	\	
							BnaA03g08110D (SUS1)	\	\	
							BnaA03g08640D (CSLA02)	Upregulated	Upregulated	
							BnaA03g09220D (Gibberellin-regulated family protein)	\	\	
							BnaA03g08500D (VAMP714)	Upregulated	Upregulated	
*qBAD.A03-2*	5,740,679-6,942,893	BA-E2, BA-E3, BA-E4, BA-BLUE, BD-E3, BD-E4, BD-BLUE	mrMLM, FarmCPU	4.36-7.27	4.29-6.34		BnaA03g13150D (GH3)	\	\	Shen et al., 2018
							BnaA03g14990D (VAMP725)	\	\	Li et al., 2017
							BnaA03g12730D (SIS)	Upregulated	Upregulated	
							BnaA03g14360D (CDC48C)	Downregulated	Downregulated	
*qBAD.A03-3*	20,456,969-21,462,759	BA-E2, BA-E3, BA-E4, BA-BLUE, BD-E4, BD-BLUE	mrMLM, FarmCPU	4.61-6.92	1.49-5.67		BnaA03g40910D (BEH1)	\	\	New
							BnaA03g40930D (GATL2)	\	\
							BnaA03g41540D (HSFA7A)	\	\
							BnaA03g42750D (CSLA10)	\	\
*qBAD.C03-2*	2,575,361-3,561,868	BA-E4, BD-E3, BD-E4, BD-BLUE	mrMLM, FarmCPU	4.45-7.70	4.55-10.94	BnaC03g06250D (LAZY1)		Downregulated	Downregulated	Liu J, et al., 2016
							BnaC03g05830D (GH3)	\	\	Sun et al., 2016
							BnaC03g05840D (GH3)	\	\	
							BnaC03g07400D (bZIP3)	\	\	
*qBAD.C04-1*	4,068,973-4,807,046	BA-E3, BA-E4, BA-E6, BD-E3, BD-BLUE	mrMLM	3.91-5.53	1.85-6.37		BnaC04g06540D (Plant calmodulin-binding protein-related)	Upregulated	Upregulated	New
*qBAD.C06-1*	22,198,524-23,400,581	BA-E3, BA-E4, BA-BLUE, BD-E3	mrMLM, FarmCPU	4.33-6.37	3.54-19.4	BnaC06g20640D (ARF17)		\	\	New
							BnaC06g20480D (GRF2)	\	\
*qBA.C07-1*	36,906,043-37,706,172	BA-E1, BA-E2, BA-E4, BA-E6, BA-BLUE	mrMLM, FarmCPU	5.08-7.03	1.72-6.29		BnaC07g34980D (NAC071)	\	Downregulated	New
							BnaC07g35000D (BSK2)	\	\
*qBD.C09-4*	39,816,234-40,792,238	BD-E3, BD-E5, BD-BLUE	mrMLM, FarmCPU	4.44-6.23	2.20-6.29		BnaC09g37040D (SUS1)	\	\	Shen et al., 2018
							BnaC09g37060D (SAUR)	\	\
							BnaC09g37090D (SAUR)	\	\
							BnaC09g37000D (TCP)	\	\

**Figure 5:**
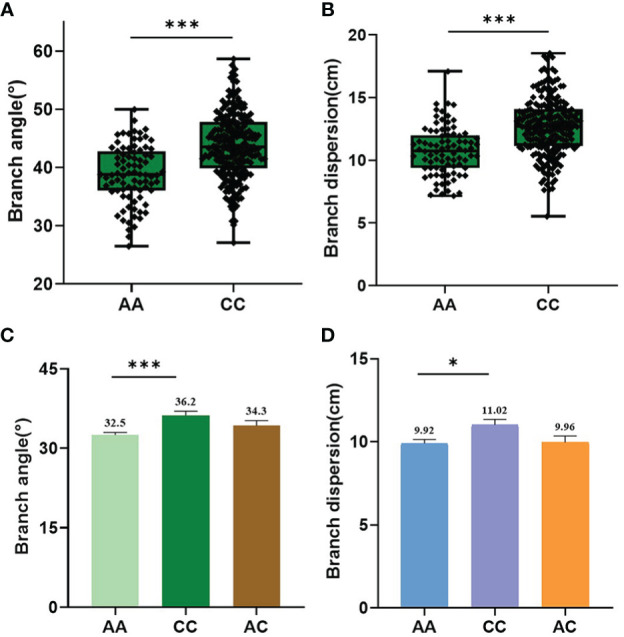
Phenotypic variation of the significant SNP chrA03_6002385 for *qBAD.A03-2*. **(A)** Haplotype analysis of chrA03_6002385 for BA in association population. **(B)** Haplotype analysis of chrA03_6002385 for BD in association population. **(C)** Haplotype analysis of chrA03_6002385 for BA in segregated progeny derived from remaining heterozygous accession 8S128. **(D)** Haplotype analysis of chrA03_6002385 for BD in segregated progeny derived from 8S128. * and *** represent the significant level at P=0.05 and 0.001 respectively, as determined by Student’s t-test.

It is worth noting that the QTL *qBAD.A03-2* explaining 6.34% of the phenotypic variation was repeatedly detected in BA-E2, BA-E4, BA-BLUE, BD-E3, BD-E4, and BD-BLUE environments (**Table 1**). Moreover, the physical interval of the *qBAD.A03-2* (5,752-6,267 kb) also overlapped with that previously reported (Li et al., 2017; Shen et al., 2018), indicating that *qBAD.A03-2* was a stable and effective QTL among different populations and environments. For *qBAD.A03-2*, the two variant bases A and C of peak SNP chrA03_6002385 accounted for 28.6% and 71.4%, respectively. BA and BD of the A allele (38.8° and 10.8 cm) were significantly smaller (P=1.10 × 10^−4^, P=1.21 × 10^−4^) than those of the C allele (43.8° and 12.7 cm) in multiple environments (**Figures 5A, B**). To further validate the phenotypic effect of *qBAD.A03-2*, a remaining heterozygous accession (8S128) with a background homozygosity of 93.42% was screened out. The BA and BD of A allele (32.6° and 9.92 cm) was significantly (P = 2.00E-4, P = 1.06E-2) smaller than those for C allele (36.2° and 11.02 cm) (**Figure 5C, D**) in its progenies. The results were generally consistent with those of GWAS, confirming that *qBAD.A03-2* was a promising QTL regulating branch architecture.

### Comparative transcriptomic analysis

To further examine the branch angle difference formation at the molecular level, a comparative transcriptomic analysis was performed. Two large branch angle accessions 8S111 (L1) and 8S092 (L2) and two small branch angle accessions 8S103 (S1) and 8S056 (S2) were investigated. Before the branch angle became stable, the cortex on the adaxial (U) and abaxial part (D) of the third branch was sampled. A total of 24 samples (LU1, LU2, SU1, SU2, LD1, LD2, SD1, and SD2) with three biological replicates per sample were used for RNA sequencing. The total reads, mapped reads, and uniquely mapped reads were shown in **Table S5**. After removing low-quality sequences, a total of 44,951,437 (91.7%)–53,306,846 (93.43%) clean reads from 24 samples were successfully mapped to the genome. Of these clean reads, 44,879,822(86.1%)–49,919,093(87.49%) were uniquely mapped, suggesting that the quality of this transcriptome sequencing data was adequate for subsequent analysis. As shown in **Figure S2**, the qRT-PCR results of four genes expression were consistent with the RNA-seq results, which confirmed the reliability of the RNA-seq.

**Figure 6:**
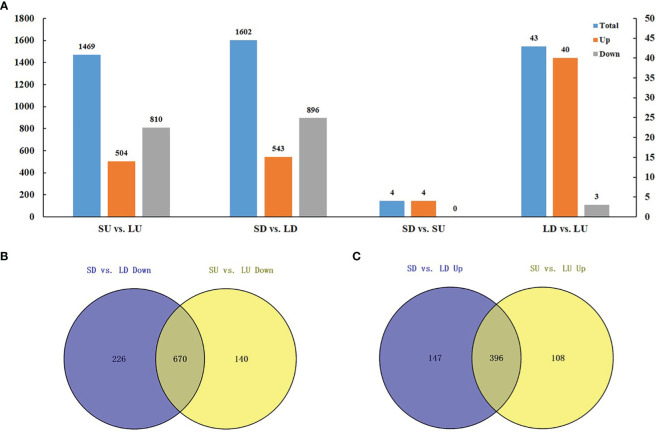
Transcriptomic analysis of branch angle in oilseed rape. **(A)** Statistical analysis of DEGs. SU vs. LU means the comparison between small- and large- branch angle accessions on the adaxial side. SD vs. LD means the comparison between small- and large- branch angle accessions on the abaxial side. SD vs. SU means the comparison between the branches adaxial and abaxial side of the small-angle accessions. LD vs. LU means the comparison between the branch’s adaxial and abaxial side of the large-angle accessions. The blue rectangle indicates all DEGs in the combination. The orange indicates consistently upregulated DEGs. The gray indicates consistently downregulated DEGs. **(B)** Upregulated DEGs between small- and large- branch angle accessions. **(C)** Downregulated DEGs between small- and large- branch angle accessions.

Two types of comparisons were performed in the identification of DEGs: (1) the comparison between the large- and small-angle accessions, namely, SU vs. LU and SD vs. LD (**Figure 6A**, **Table S6**). (2) the comparison between the adaxial and abaxial side of branches, namely, LU vs. LD and SU vs. SD (**Figure 6A**, **Table S6**). SU vs. LU meant the comparison between small- and large- branch angle accessions on the adaxial side. Similarly, SD vs. LD indicated the comparison between small- and large- branch angle accessions on the abaxial side.

In total, 1469 DEGs were identified in the comparison of SU vs. LU, of which 1314 were consistently up- (504) or downregulated (810) in four combinations, accounting for a high proportion of 89.4% (**Figure 6A**). In addition, 1602 DEGs were identified in the comparison of SD vs. LD, of which 1439 DEGs were consistently up- (543) or downregulated (896) in the four combinations, accounting for 89.8% (**Figure 6A**). It should be noted that most of the common DEGs in different combinations show consistent upregulation or downregulation in S vs. L, indicating a ubiquitous regulation mechanism for branch angles. Moreover, among the DEGs in the small- and large- branch angle accessions, 670 DEGs were simultaneously downregulated in adaxial and abaxial sides, accounting for 74.8% (670/896) and 82.7% (670/810), respectively (**Figure 6B**), and the 396 DEGs were simultaneously upregulated, accounting for 73.0% (396/543) and 82.7% (396/504), respectively (**Figure 6C**). These results showed that most DEGs in the small- and large- branch angle accessions were co-expressed on the adaxial and abaxial side of the branch.

In addition, SU vs. SD represented a comparison between the branches adaxial and abaxial side of the small-angle accession (**Figure 6A**; **Table S6**). Similarly, LU vs. LD indicated a comparison between the branches adaxial and abaxial side of the large-angle accession (**Figure 6A**; **Table S6**). Four DEGs were identified in SU vs. SD (4 upregulated), and 43 DEGs in LU vs. LD (40 upregulated and 3 downregulated) (**Figure 6A**; **Table S6**). These results suggested that fewer DEGs were found in the adaxial and abaxial sides of the branch.

**Figure 7:**
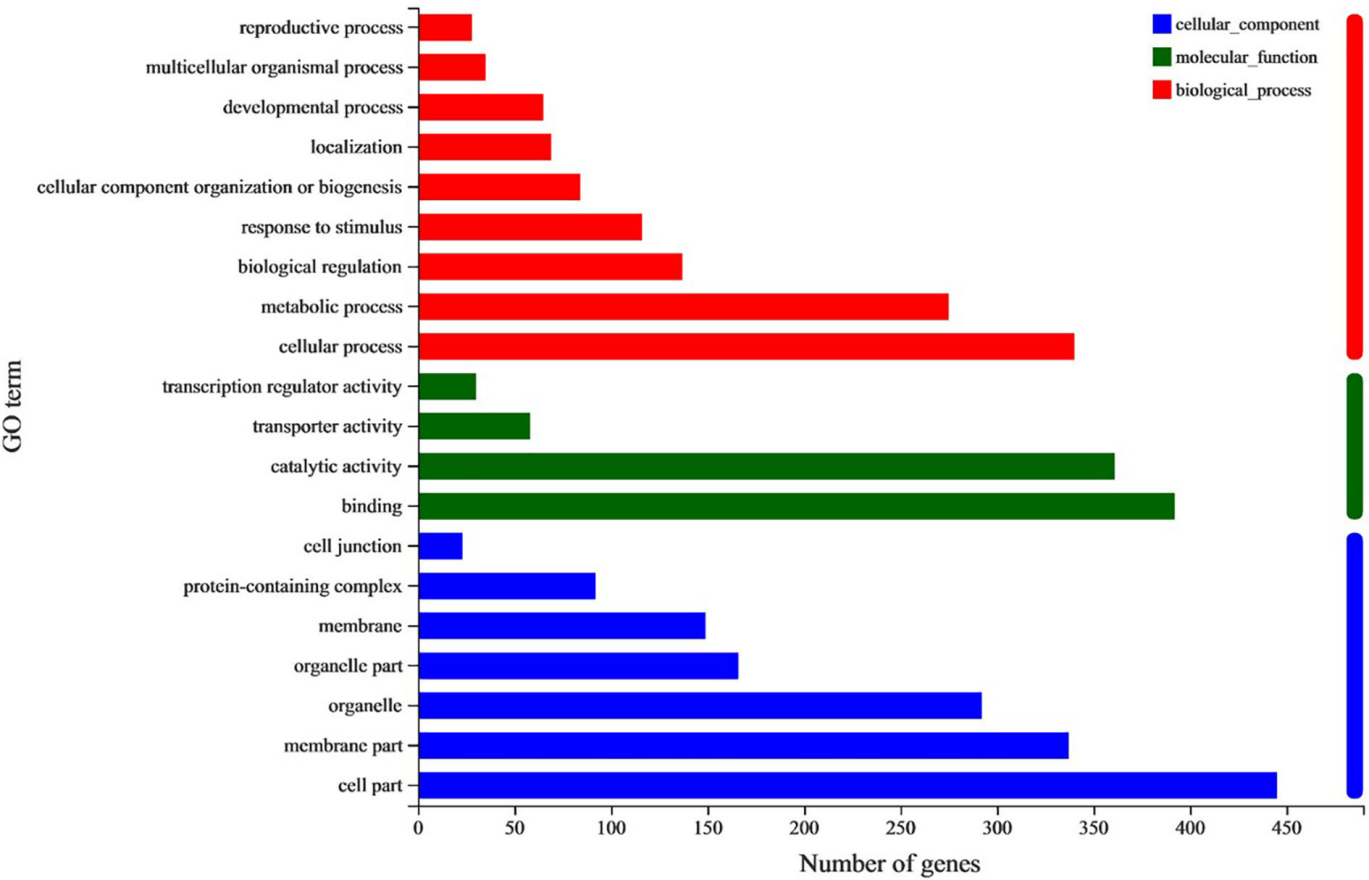
GO analysis of common DEGs between SU vs. LU and SD vs. LD. The horizontal and vertical axes show the number and enriched class of DEGs. Red columns, Biological process; Green columns, Molecular function; Blue columns, Cellular component.

A total of 1066 common DEGs between both large- and small- angle accessions in branches adaxial and abaxial sides were selected for GO enrichment analysis. The top 20 significantly enriched GO terms were shown in **Figure 7**. For the cellular component category, most DEGs were mainly annotated to the cell membrane and organelle part. For the biological process category, most DEGs were mainly assigned to cellular component organization or biogenesis, metabolic process, and the developmental and reproductive process. For the molecular function category, these DEGs were primarily annotated to binding, catalytic activity, transporter, and transcription regulator activity. KEGG enrichment analysis was performed to further determine the metabolic pathways in which DEGs were enriched. The results showed that the DEGs were significantly enriched in fatty acid elongation, other glycan degradation, and indole alkaloid biosynthesis pathways (**Table S7**).

### Identification of candidate genes in 10 stable association loci

By comparing the physical positions of homologs of branch angle genes and the physical intervals of 10 stable association loci, a total of four genes were identified as direct candidates (**Table 1**). In addition, 32 genes that were predicted to be related to the regulation of branch/tiller angle were defined as indirect candidates. More importantly, 9 DEGs were localized in the genomic intervals of these association loci, which were considered as potential candidates. It should be noted that some candidate genes were not only functionally related but also differentially expressed, and thus they were promising candidates. For example, *BnaC03g06250D* was the direct candidate in the major association loci *qBAD.C03-2*. This gene was homologous to the branch angle gene *LAZY1*, and it was downregulated in both adaxial and abaxial sides in large- vs. small-angle accessions. For another example, *BnaA03g08500D* was the candidate gene in the major association loci *qBAD.A03-1*, and it was the homolog of *VAMP714* which was mainly responsible for polar auxin transport and affected the gravitropism of the branch. Moreover, *BnaA03g08500D* was significantly upregulated in large- vs. small-angle accessions. For the validated major association loci *qBAD.A03-2*, no known BA genes were found in its genomic interval, suggesting that there might exist a new gene related to branch architecture in this loci in oilseed rape.

## Discussion

### Characterization of branch angle and branch dispersion

The compact branch architecture optimizes rapeseed canopy and is crucial for population photosynthesis. The existing studies tend to evaluate the compact degree by the branch angle (Sun et al., 2016; Li et al., 2017). In oilseed rape, branch angle is measured based on the fitting line in basal, which can not accurately reflect the compact degree since branch is not always grown in a fixed direction along a straight line. To better characterize the branch architecture, we introduced a new index branch dispersion degree (BD) for the first time, which could reflect the dispersion degree of the upper part of the branch. The average length of branches in our association panel was about 50 cm, and thus BD was defined as the distance between the branch and stem at the point 25 cm (about the middle site of branches) far from the branch junction. The high correlation of BD among three biological replicates (r = 0.82, 0.81, and 0.81) confirmed it to be a feasible and repeatable approach for evaluating the compactness of branch architecture, casting light on later studies.

### Relationship between BA and BD

At the phenotypic level, BA and BD showed a significant positive correlation, suggesting that the two traits were related to each other. At the genetic level, nearly one-fifth of the association loci were responsible for both traits, which was in line with their phenotypic correlation, whereas the remaining four-fifths of association loci were only responsible for one trait, indicating that the genetic control over BA and BD was largely different. The possible reason might be that BA represents the branching degree in the early stage, which is mainly determined by genetic factors, whereas BD reflects the final branching degree in the stable stage, which is influenced by multiple factors such as genetics, environment, and gravitropism. These results were highly accordant with our observation of branching dynamic process, suggesting a stage-specific regulation of branch architecture in oilseed rape.

### Comparison of QTLs responsible for branch architecture in *B. napus*

MLM has been widely adopted in GWAS for a variety of agronomic traits. However, MLM may overcompensate for population structure and kinship, which can result in false negatives (Yu et al., 2006). Consistently, only five significant SNPs were detected in 9 trait-environment combinations by MLM, which were much less than those detected by the other two models. For example, the peak SNP chrA03_6002385 was not detected by MLM, but this SNP reached the significance threshold in both mrMLM and FarmCPU in multiple environments. The mrMLM is modified from MLM and has the advantage of rapidly screening with a slightly less stringent criterion than *Bonferroni* in MLM, which might explain the much more target trait-related SNPs (146) detected by mrMLM than by MLM (Wang S. B. et al., 2016). FarmCPU, a recently developed model, not only provides strong statistical power but also good control over false positives and negatives (Yin et al., 2021), which was supported by our results that FarmCPU detected more significant robust SNPs.

To discover the branch architecture QTLs, three models were adopted in this study. The 10 association loci were identified as the ones highly related to target traits when different models, environments, and traits were taken into consideration, 5 of which were found to be overlapped with those reported in previous studies, and the other 5 were newly identified, suggesting the feasibility and reliability of multiple factor-based GWAS strategy. For example, QTL *qBAD.A03-2* was detected by both mrMLM and FarmCPU models in multiple environments across BA and BD traits. Haplotype analysis of *qBAD.A03-2* showed that the phenotype corresponding to genotype A was consistently smaller than that corresponding to genotype C in multiple environments (**Figure 5**). Moreover, *qBAD.A03-2* was further verified using a secondary segregation population derived from the remaining heterozygous line 8S128, indicating that this locus was a real QTL responsible for BA and BD.

### Direct candidate genes in ten stable association loci

Four genes localized in the genomic intervals of 10 stable association loci were homologous to the known BA genes, and therefore they were considered as direct candidates. Of these 4 genes, *BnaC03g06250D* was the homolog of *LAZY1* which played an important role in the asymmetric distribution of auxin. The RNAi lines of *AtLAZY1* showed a larger branch angle in *Arabidopsis* (Yoshihara and Iino, 2007; Yoshihara et al., 2013). Highly accordant with this report, our results showed that *BnaC03g06250D* was one upregulated DEG in both adaxial and abaxial sides in small- vs. large- branch angle accessions. In addition, microRNAs (miRNAs) have been reported to regulate the tiller angle in rice, and the overexpression of miRNA167a in rice suppressed the expression of its target gene *OsARF17*, thus resulting in the asymmetric distribution of auxin, finally causing the phenotype of dwarfism and larger tiller angle (Li Y. et al., 2020). As the homolog of *OsARF17*, *BnaC06g20640D* might be involved in regulating branch angle in oilseed rape. The polar auxin transport (PAT) plays a vital role in the formation and variation of branch angle. PAT is mediated by different auxin carrier vector proteins such as PIN transporter proteins (Adamowski and Friml, 2015). *BnaA02g16500D* and *BnaA03g10430D* were the homologs of *PIN1* and *PIN2*, respectively. The inhibition of *OsPIN1* can change the polar auxin transport, thus increasing the tiller angle in rice (Xu et al., 2005). The overexpression of *OsPIN2* in rice can increase the tiller angle by directly promoting PAT. Interestingly, in *OsPIN2-*overexpressing plants, *OsLAZY1* expression is significantly suppressed (Chen et al., 2012). It should be noted that all four direct candidates in our detected association loci were related to the asymmetric distribution of auxin, which highlighted the importance of the auxin pathway in regulating the natural variation of branch angle in oilseed rape germplasm. The other three direct candidates except for *BnaC03g06250D* (*LAZY1*) exhibited no expression difference in the small- vs. large-angle accessions. However, all four candidates displayed sequence variations against the reported sequence database of *B. napus* (http://yanglab.hzau.edu.cn/BnVIR). Therefore, future studies are suggested to develop molecular markers based on the sequence variations of these four direct candidates to identify functional genes.

## Data availability statement

The datasets presented in this study can be found in online repositories. The names of the repository/repositories and accession number(s) can be found in the article/**Supplementary Material**.

## Author contributions

HW, XW, XD, and JD designed the research. YW, KW, TA, ZT, and JD participated in the population phenotype investigation; YW conducted the data analysis and wrote the manuscript. YW, JS, and JD revised the manuscript. All authors read and approved the final manuscript. All authors contributed to the article and approved the submitted version.

## Funding

This research was supported by the Wuhan Science and Technology Major Project on Key techniques of biological breeding and Breeding of new varieties (2022021302024851), the science and technology major program of Hubei Province (2021ABA011), the International Science & Technology Innovation Program of Chinese Academy of Agricultural Sciences (CAAS-ZDRW202105), the Agriculture Research System of MOF and MARA of China (CARS-13), the Agricultural Science and Technology Innovation Project (CAAS-ASTIP-2013-OCRI), the Agricultural Science and Technology Innovation Program (ASTIP No. CAAS-ZDRW202201).

## Acknowledgments

The authors thank Xiya Zhang and Yujiao Liu for their help in the fieldwork.

## Conflict of interest

The authors declare that the research was conducted in the absence of any commercial or financial relationships that could be construed as a potential conflict of interest.

## Publisher’s note

All claims expressed in this article are solely those of the authors and do not necessarily represent those of their affiliated organizations, or those of the publisher, the editors and the reviewers. Any product that may be evaluated in this article, or claim that may be made by its manufacturer, is not guaranteed or endorsed by the publisher.

## Supplementary material

Supplementary Figure 1 Schematic diagram of BA and BD trait examination.

Supplementary Figure 2 The qRT-PCR validation of four random DEGs. The horizontal axes represent eight samples of transcriptomic analysis. The blue and orange vertical axes represent the FPKM values and relative expression levels, respectively.
