# Supplementary figures and images for: Genetic dissection of branch architecture in oilseed rape (Brassica napus L.) germplasm
Source: Front Plant Sci. 2022 Oct 28;13:1053459. doi: 10.3389/fpls.2022.1053459 (PMC9650407; doi:10.3389/fpls.2022.1053459)

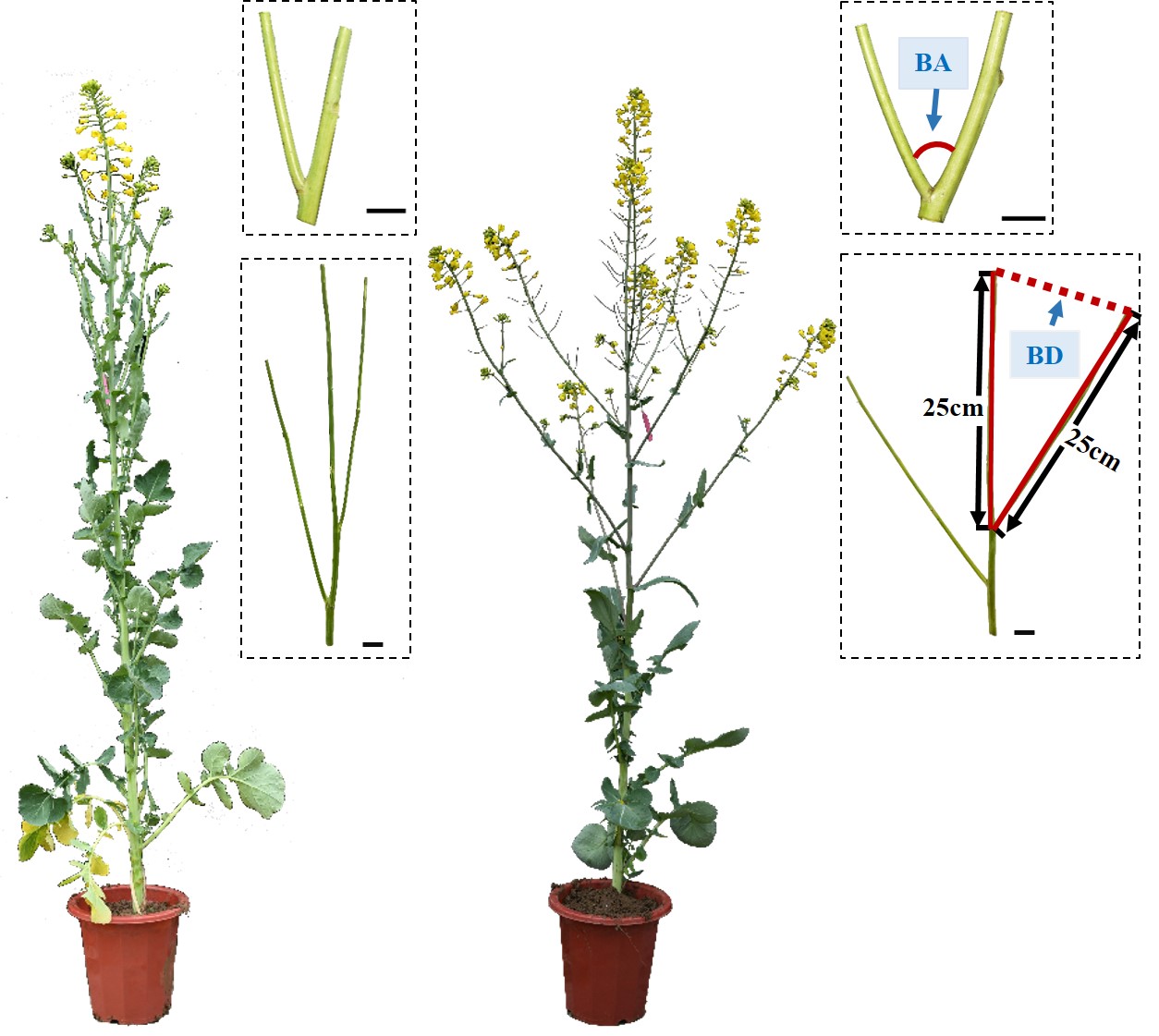

Supplement: Supplementary Figure 1 — Schematic diagram of BA and BD trait examination. [file Image_1.jpeg]

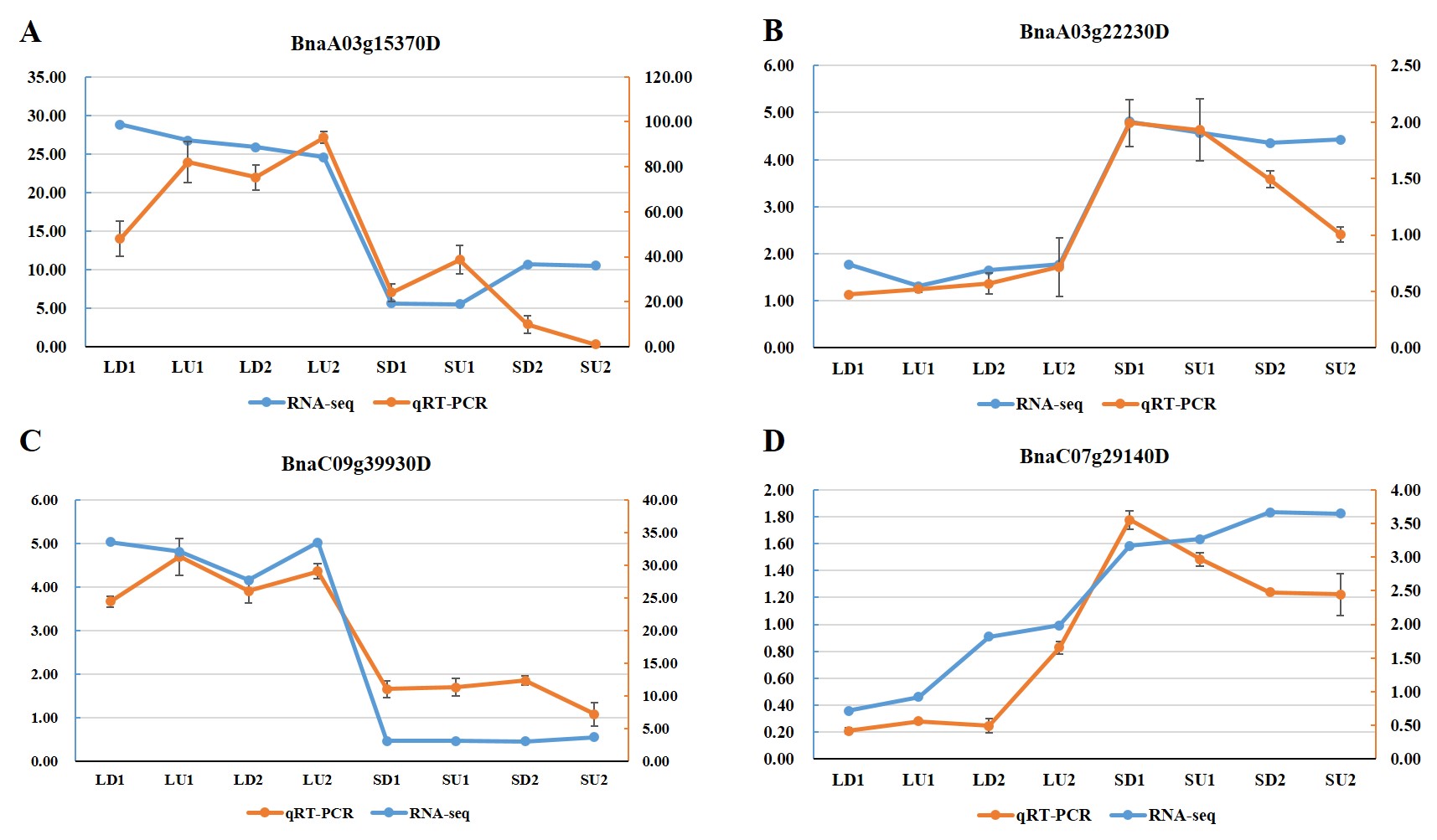

Supplement: Supplementary Figure 2 — The qRT-PCR validation of four random DEGs. The horizontal axes represent eight samples of transcriptomic analysis. The blue and orange vertical axes represent the FPKM values and relative expression levels, respectively. [file Image_2.jpeg]
